# Shikonin induces ferroptosis in multiple myeloma *via* GOT1-mediated ferritinophagy

**DOI:** 10.3389/fonc.2022.1025067

**Published:** 2022-10-25

**Authors:** Wenxia Li, Hangjie Fu, Liuyuan Fang, Hui Chai, Tianwen Gao, Zhenzhen Chen, Shenxian Qian

**Affiliations:** ^1^ The Fourth School of Clinical Medicine, Zhejiang Chinese Medical University, Hangzhou, China; ^2^ Department of Hematology, Affiliated Hangzhou First People’s Hospital, Zhejiang University School of Medicine, Hangzhou, China; ^3^ College of Life Science, Zhejiang Chinese Medical University, Hangzhou, China

**Keywords:** multiple myeloma, shikonin, ferroptosis, ferritinophagy, GOT1

## Abstract

Multiple myeloma (MM) is an incurable hematological malignancy that lacks effective therapeutic interventions. Ferroptosis is a newly discovered form of cell death that has shown great potential for MM therapy. As a proteasome inhibitor and necroptosis inducer, shikonin (SHK) performs dual functions in MM cells. However, whether SHK inhibits the development of MM *via* ferroptosis or any other mechanism remains elusive. Here, we provide evidence that SHK treatment was capable of inducing ferroptosis and immunogenic cell death (ICD) in MM. The results showed that SHK treatment induced lactate dehydrogenase release, triggered cell death, evoked oxidative stress, and enhanced ferrous iron and lipid peroxidation levels. Furthermore, treatment with ferroptosis inhibitors reversed SHK-induced cell death, which indicated that ferroptosis contributed to this phenomenon. Meanwhile, ferroptosis was accompanied by the extracellular release of Adenosine 5’-triphosphate (ATP) and High mobility group protein B1 (HMGB1), which are characteristics of ICD. Further investigation showed that glutamic-oxaloacetic transaminase 1 (GOT1) acted as a critical mediator of SHK-induced ferroptosis by promoting ferritinophagy. In conclusion, our findings suggest that SHK exerts ferroptotic effects on MM by regulating GOT1-mediated ferritinophagy. Thus, SHK is a potential therapeutic agent for MM.

## Introduction

Multiple myeloma (MM) is a hematological malignancy of plasma cells, characterized by substantial intraclonal genetic heterogeneity and symptoms such as anemia, bone lesions, hypercalcemia, and impairment of renal function (1). With the continuous advent of new drugs and improvements in detection methods, MM treatment has been remarkably improved. However, MM remains incurable (2). Therefore, it is imperative to identify new therapeutic targets and pharmaceuticals.

Ferroptosis is a newly discovered form of regulated cell death mediated by intracellular iron (3). Abnormal iron metabolism, lipid peroxidation, and weakened antioxidant systems are hallmarks of ferroptosis (4). Ferroptosis is inhibited by deferoxamine mesylate (DFOM), an iron chelator, and liproxstatin-1 (Lip-1), an antioxidant. Recently, multiple studies have demonstrated that ferritinophagy, a novel type of cell autophagy, can trigger cellular ferroptosis, as its overactivation causes intracellular iron overload (5–7). Ferritinophagy is characterized by the degradation of ferritin and the release of Fe^2+^. Generally, ferritin degradation maintains iron homeostasis in cells. However, this process is excessively activated under pathological conditions, resulting in an imbalance in the iron content of cells (8). A recent study reported that glutamic-oxaloacetic transaminase 1 (GOT1) might be involved in ferritinophagy (9). GOT1, which is located in the cytoplasm, catalyzes the formation of NADH and transfers reducing equivalents from the cytosol into the mitochondria. As an essential factor for oxidative phosphorylation and energy generation in cells, GOT1 is a highly important therapeutic target (10, 11). Therefore, the discovery of anti-GOT1 components in natural products could provide novel pharmaceutical candidates.

Shikonin (SHK), a naphthoquinone, is a major bioactive component present in the roots of *Lithospermum erythrorhizon* and exhibits strong antitumor activities. A previous study reported that SHK can inhibit proteasomes and induce necroptosis in MM cells (12). Furthermore, SHK can induce Bcr/Abl-positive chronic myelogenous leukemia cell apoptosis and renal cancer cell autophagy *via* reactive oxygen species (ROS) generation (13, 14), in addition to promoting necroptosis in sunitinib-resistant renal cell carcinoma, glioma, docetaxel-resistant prostate cancer, and non-small cell lung cancer (15–17). Additionally, SHK inhibits the growth of esophageal squamous cell carcinoma and hepatocellular carcinoma cells by regulating glucose metabolism *via PKM2* (18, 19). However, whether SHK inhibits the development of MM *via* ferroptosis or any other mechanism remains largely unknown.

The PharmMapper database is an online tool for potential drug target identification by reversed pharmacophore matching the query compound against an in-house pharmacophore model database (20). FerrDb, a database for regulators and markers of ferroptosis and ferroptosis-disease associations (21). We used this database to explore the potential targets of SHK-induced ferroptosis in MM. Molecular docking technology is based on the “lock and key principle” of ligand-receptor interaction and uses computer-aided drug design to conduct virtual drug screening, which is a quick and effective way to identify drug targets (22). Here, we studied the docking of key hub protein to predict the interaction between SHK and its predicted target. The GEO (gene expression omnibus) database is based on gene chip technology and can be used to construct disease validation sets. We used the GEO database to obtain clinical data and evaluate the correlation between the key target and the disease.

In short, this study aimed to investigate whether SHK has an anti-tumor effect on MM *in vitro* and in a xenograft mouse model *in vivo* and elucidate the mechanisms involved.

## Materials and methods

### Reagents

SHK, chloroquine (CQ), and Lip-1 were purchased from Selleck Chemicals (Houston, TX, USA). DFOM was purchased from MedChem Express (Shanghai, China). The stimulus concentrations were as follows: CQ, 10 μM; Lip-1, 10 μM; and DFOM, 25 μM.

### Cell Culture and cell line-derived xenograft mouse model

The human MM cell lines RPMI 8226 and U266 were kindly gifted by Dr. Wenbin Qian (Institute of Hematology, Zhejiang University, Hangzhou, China) and cultured in RPMI 1640 medium containing 10% fetal bovine serum at 37°C in a Forma series II water-jacketed CO_2_ incubator (Thermo Fisher Scientific, Waltham, MA, United States). Both cell lines were used for the *in vitro* studies.

Six 5-week-old female Nod-SCID mice were used in the *in vivo* study. The animal experiment was conducted at the Zhejiang Chinese Medical University Laboratory Animal Research Center, with certification (approval number: IACUC-20211220-04). Mice were allowed free access to food and water under specific-pathogen-free (SPF) conditions at 22°C with 12 h of abundant light every day. The mice were allowed to acclimatize for 7 d and then were injected with 1 × 10^7^ RPMI 8226 cells. The weights and tumor sizes of the mice were assessed, and the tumor volumes (V) were calculated every 2 d using the following formula: (maximum length) × (maximum width)^2^/2. As the tumors were detectable, the mice were stochastically divided into the control (intraperitoneally injected with phosphate-buffered saline (PBS) every 2 d) and treatment groups (intraperitoneally injected with 4 mg/kg SHK in PBS buffer every 2 d). After 10 d, the mice were sacrificed, and tumors were collected for further analysis.

### Cell viability assay

The methylthiazolyldiphenyl-tetrazolium bromide (MTT) (Beyotime, Shanghai, China) and lactate dehydrogenase (LDH) assays were used to evaluate MM cell proliferation and cytotoxicity. A total of 2 × 10^4^ cells/well were seeded in a 96-well plate, and a series of increasing concentrations of SHK or ferroptosis inhibitors was added to the wells; the plate was then incubated in a 37°C incubator (5% CO_2_) for 24 h. Finally, 20 μL MTT solution (5 mg/mL) was added to each well. The plate was then incubated at 37°C for 3 h before measuring the absorbance of each well at 570 nm using FLUOstar Omega plate reader (BMG Labtech, Offenburg, Germany). The extent of cellular injury was determined by the amount of LDH released into the medium using an LDH cytotoxicity detection kit (Beyotime) according to the manufacturer’s instructions.

### Adenosine 5’-triphosphate (ATP) detection

MM cells were seeded in six-well plates and treated with and without 1 μmol/L SHK for 24 hours. The supernatants were then collected to measure ATP release. Cells were lysed to measure the cellular ATP level. ATP was quantified using the Enhanced ATP Assay Kit (Beyotime), according to the manufacturer’s instructions. SpectraMax M3 multifunctional microplate reader (Molecular Devices, San Jose, CA, USA) was applied for ATP quantification, and the amount of luminescence was reported as fold change in relative luminescent units (RLUs).

### Cell apoptosis determination

Cell apoptosis was determined using the Annexin V- fluorescein isothiocyanate (FITC) Apoptosis Detection Kit (Beyotime) according to the manufacturer’s instructions. Briefly, 5 × 10^5^ cells/well were seeded in a 6-well plate, and each well was treated with SHK (500 and 1000 nmol/mL). After 24 h, the cells in different wells were separated, collected with PBS buffer, and stained with annexin V- (FITC) and propidium iodide (PI) at 23–25°C in the dark for 15 min. Cells were processed using a BD Accuri C6 flow cytometer (BD Biosciences, San Jose, CA, United States) and analyzed using FlowJo software (10.0.7r2 version).

### Determination of oxidative stress-related indicators

The levels of malondialdehyde (MDA), superoxide dismutase (SOD), and glutathione (GSH) in cells were determined using a commercial MDA assay kit (Beyotime), SOD assay kit (Nanjing Jiancheng, China), and GSH assay kit (Beyotime), respectively, according to the respective manufacturer’s protocols.

### Ferrous iron detection

Cellular and mitochondrial ferrous iron concentrations were assessed using the FerroOrange probe (Dojindo, Japan) and Mito-FerroGreen probe (Dojindo), respectively. Briefly, 5 × 10^5^ cells/well were seeded in 6-well plates and treated with 1 μmol/L SHK with or without DFOM. After 24 h, 1 μmol/L FerroOrange and 2 μmol/L Mito-FerroGreen probes were added to the cell culture, and the plates were incubated at 37°C for 30 min. The stained cells were monitored using a BD Accuri C6 flow cytometer (BD Biosciences). The tissue ferrous iron and total iron concentrations were assessed using an iron assay kit (Dojindo) according to the manufacturer’s instructions.

### Cellular lipid ROS detection

Cells were plated at a density of 5 × 10^5^ cells/well in a 6-well plate. Then, 1 μmol/L SHK, with or without DFOM, was added to the wells. After 24 h, 2 μmol/L BODIPY-581/591 C11 (Thermo Fisher Scientific) was added to the cell culture, and the plate was incubated at 37°C for 30 min to stain lipid ROS in the cells. Thereafter, the cells were collected and washed twice with PBS, followed by resuspension in 500 μL PBS. The cells were monitored with a BD Accuri C6 flow cytometer (BD Biosciences) and analyzed using the software FlowJo (10.0.7r2 version).

### Cellular mitochondrial lipid ROS assay

The RPMI 8226 cells with and without the SHK treatment were incubated with 0.1 mol/L MitoPeDPP (Dojindo) at 37°C for 15 min. The cells were then washed twice. The cells were then examined using the Laser scanning confocal microscope (Zeiss, Oberkochen, Germany).

### High mobility group protein B1 (HMGB1) release assay

MM cells were incubated in six-well plates and treated with 1 μmol/L SHK. After 24 hours of incubation, the supernatants were obtained for the measurement of HMGB1 concentration by Human HMGB1 ELISA Kit (Jiangsu Jingmei Biological Technology Co., Ltd, Yancheng, China) as described by the manufacturer. The absorbance value was measured at 450 nm using a SpectraMax M3 multifunctional microplate reader (Molecular Devices).

### RNA extraction and quantitative reverse transcription-PCR

Total RNA was isolated using the RNAeasy™ kit (Beyotime) according to a previously described protocol (23). The isolated RNA was quantified *via* the photometric method using a NanoDrop (Thermo Fisher Scientific). Next, 1 μg total RNA was reverse-transcribed into cDNA using the HiFiScript cDNA synthesis kit (Cwbio, Taizhou, China) according to the manufacturer’s recommendations. All primers used in this study were synthesized by Sangon Biotech (Shanghai, China). The primer sequences are listed in **Table 1**. qRT–PCR was performed using the UltraSYBR mixture (Cwbio) and ABI 7500 real-time PCR detection system (Thermo Fisher Scientific) to obtain the comparative threshold cycle (Ct) value of the target genes. *β-actin* served as an internal control for qRT-PCR. The relative fold change in gene expression was calculated using the 2^-(ΔΔCt)^ method.

**Table 1:** Primer sets used for qPCR.

Gene (Homo)	Forward primer (5’-3’)	Reverse primer (5’-3’)
*GPX4*	ATGGTTAACCTGGACAAGTACC	GACGAGCTGAGTGTAGTTTACT
*SLC7A11*	TTACCAGCTTTTGTACGAGTCT	GTGAGCTTGCAAAAGGTTAAGA
*β-Actin*	CACGATGGAGGGGCCGGACTCATC	TAAAGACCTCTATGCCAACACAGT

### Western blot analysis

Cells were lysed in a radioimmunoprecipitation (RIPA) buffer with protease inhibitors and phosphatase inhibitors (CWBIO), and the lysate was centrifuged at 12000 g for 15 min to obtain the supernatant containing the proteins. Protein concentration was determined using a bicinchoninic acid kit (Beyotime). The proteins were then separated *via* 10% or 12% sodium dodecyl sulfate-polyacrylamide gel electrophoresis (SDS-PAGE) and electrotransferred to a polyvinylidene difluoride (PVDF) membrane (Millipore, Billerica, MA, USA). The membrane was blocked with 5% nonfat milk for 1 h and incubated with anti-GPX4 (1:1000, bm5231, Boster), anti-SLC7A11 (1:1000, bm5318, Boster), anti-GOT1 (1:1000, bm5408, Boster), anti-FTH1 (1:1000, bm5356, Boster), anti-NCOA4 (1:250, sc-373739, Santa Cruz), anti-ATG5 (1:1000, ab108327, Abcam), anti-ATG7 (1:1000, a00346, Boster), anti-ATG16L1 (1:1000, 8089S, Cell Signaling Technology), anti-SQSTM1 (1:1000, pb0468, Boster), anti-LC3B (1:1000, ab51520, Abcam), anti-GAPDH (1:10000, ET1601-4, Huabio), and anti-*β-actin* antibodies (1:10000, ac026, Abclonal) at 4°C. After 12 h, the membranes were washed three times with Tris-buffered saline containing 0.1% Tween 20, incubated with the corresponding secondary antibody, and observed under ChemiScope Series (Clinx Science instrument Co. Ltd, Shanghai, China) using an enhanced chemiluminescence kit (Vazyme, Nanjing, China).

### Target prediction

The chemical structure of SHK was downloaded from the PubChem Compound Database (https://www.ncbi.nlm.nih.gov/pccompound) and uploaded to PharmMapper (http://lilab-ecust.cn/pharmmapper/, 2017 version) to obtain the predicted targets. Targets with norm-fit scores were assessed. Ferroptosis-related gene data were downloaded from the FerrDb database (http://www.zhounan.org/ferrdb). Finally, specific targets of SHK involved in ferroptosis regulation were selected using the BioinfoGP (https://bioinfogp.cnb.csic.es/tools/venny/) platform, an online tool.

### Molecular docking

The two-dimensional structure of SHK was imported into Chem3D (CambridgeSoft Ltd. Co., USA) to establish the three-dimensional structure and obtain the optimized energy. Thereafter, AutoDockTools-1.5.6 (The Scripps Research Institute) was employed in the file trans-format. Simultaneously, the crystal structure of the target protein was obtained from the PDB database (https://www.rcsb.org/), which was modified using PyMOL (Schrodinger Co., USA) to delete conserved water molecules and hetero-molecules, and then imported into AutoDockTools-1.5.6 to add hydrogen atoms and identify their active pockets, which were also saved in the pdbqt format. Finally, SHK was docked to the target protein. The docking results were analyzed and represented using PyMOL. The docking effect of the molecule was evaluated and is shown as an affinity value.

### Clinical data collection

The GSE39754, GSE6477, GSE4452, and GSE4581 datasets from the Gene Expression Omnibus (http://www.ncbi.nlm.nih.gov/geo) were used in this study. Gene expression information and related clinical information was collected from 21 healthy donors, 22 patients with monoclonal gammopathy of undetermined significance (MGUS), 24 patients with smoldering multiple myeloma (SMM), and 271 patients with MM.

### Survival analysis

We downloaded clinical and prognostic information from GSE4452 and subsequently performed Kaplan–Meier (K–M) estimator analysis to assess the prognostic role of *GOT1* in patients with MM. We also performed K–M survival analysis on GSE4581 to verify whether *GOT1* is essential for prognosis.

### Immunohistochemical staining

Xenograft tumor tissue samples from Nod-SCID mice were collected, fixed in 4% paraformaldehyde, and embedded in paraffin. The embedded samples were sliced into 4 μm-thin sections, which were deparaffinized, dehydrated, and subjected to antigen retrieval by immersing in a citrate buffer at 98°C for 15 min to block endogenous peroxidase activity. Thereafter, samples were subjected to H_2_O_2_ treatment and blocked with a 3% bovine serum albumin (BSA) solution for 30 min. The samples were then hybridized with antibodies against glutathione peroxidase 4 (GPX4; 1:100, bm5231, Boster), GOT1 (1:100, bm5408, Boster), LC3B (1:2000, ab51520, Abcam), FTH1 (1:100, bm5356, Boster), NCOA4 (1:200, a5695, Abclonal), or 4HNE (1:25, ab51520, Abcam). After 12 h, the slices were incubated with the corresponding secondary antibodies and then subjected to staining using a 3, 3’-diaminobenzidine (DAB) staining kit (Vector Laboratories, USA).

### Statistical analysis

The results are expressed as mean ± standard deviation (SD). Two-tailed unpaired *t*-tests and a one-way analysis of variance were used for statistical analyses. All statistical analyses were carried out using GraphPad Prism. Statistical significance was set at p < 0.05.

## Results

### SHK treatment triggers cell death and evokes oxidative stress

**Figure 1:**
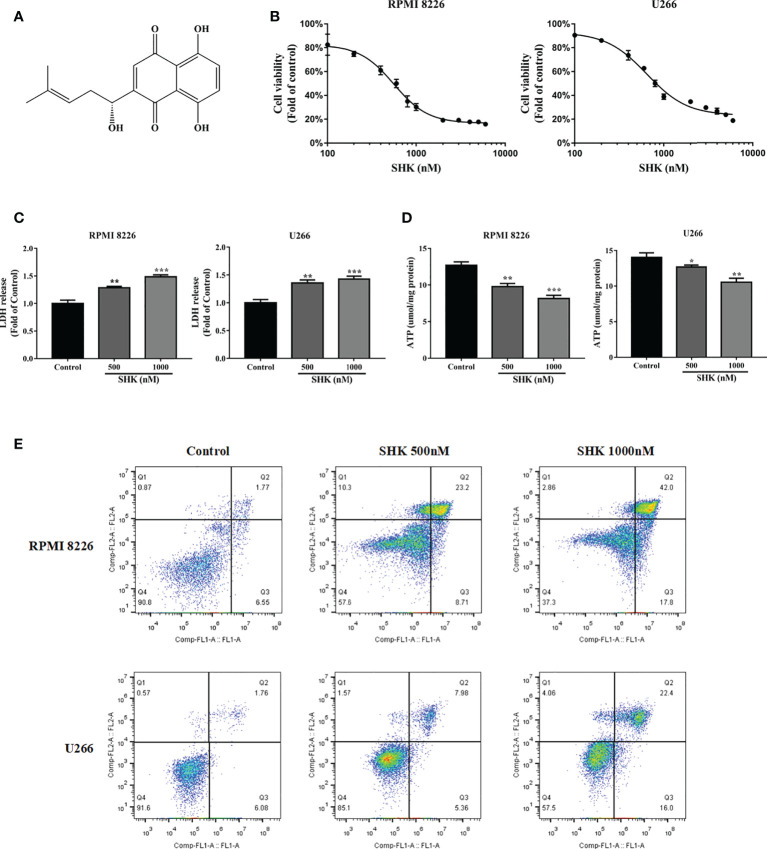
SHK has anti-myeloma effects. **(A)** The chemical structure of SHK. **(B)** MTT to test the inhibitory effect of SHK on MM cell proliferation. **(C)** LDH release levels in MM cells treated with SHK. **(D)** ATP levels in MM cells treated with SHK. **(E)** MM cells were incubated with SHK or vehicle for 24 h, then stained with annexin V-FITC/PI and analyzed by flow cytometry. Data are presented as the mean ± SD. *p < 0.05, **p < 0.01, ***p < 0.001.

The chemical structure of SHK is shown in **Figure 1A**. To explore the antitumor potential of SHK in MM, the human MM cell lines RPMI 8226 and U266 were treated with increasing concentrations of SHK, and cell viability was detected *via* the MTT assay. The results are shown in **Figure 1B**. SHK treatment reduced cell viability in a dose-dependent manner; meanwhile, LDH release increased (**Figure 1C**) and cellular ATP level decreased (**Figure 1D**). SHK-triggered MM cell death was observed using annexin V-FITC/PI staining. A markedly high percentage of dead cells was observed in both the RPMI 8226 and U266 cells in annexin V-FITC/PI staining assays (**Figure 1E**). These data indicate that SHK treatment may effectively suppress the development of MM. Next, we evaluated the stimulatory effects of SHK on oxidative stress *in vitro* and found that SHK treatment led to an increase in MDA content in cells. In contrast, the levels of SOD and GSH decreased in cells (**Table 2**).

**Table 2:** Effects of SHK on MDA, SOD and GSH levels in MM cells.

Cell	Group	MDA (μmol/mg protein)	SOD (U/mg protein)	GSH (μmol/mg protein)
RPMI 8226	Control	1.58 ± 0.07	8.64 ± 0.19	7.11 ± 0.15
	SHK 500 nM	2.71 ± 0.20 ***	7.35 ± 0.18**	5.06 ± 0.19 ***
	SHK 1000 nM	4.22 ± 0.18 ***	4.85 ± 0.24***	3.43 ± 0.06 ***
U266	Control	1.14 ± 0.14	10.36 ± 0.17	9.76 ± 0.76
	SHK 500 nM	2.97 ± 0.25 ***	8.91± 0.09***	7.06 ± 0.17 **
	SHK 1000 nM	5.32 ± 0.52 ***	6.20± 0.63***	3.78 ± 0.17 ***

Data are presented as the mean ± SD. **p < 0.01, ***p < 0.001.

### SHK treatment leads to the ferroptosis of MM cells

**Figure 2:**
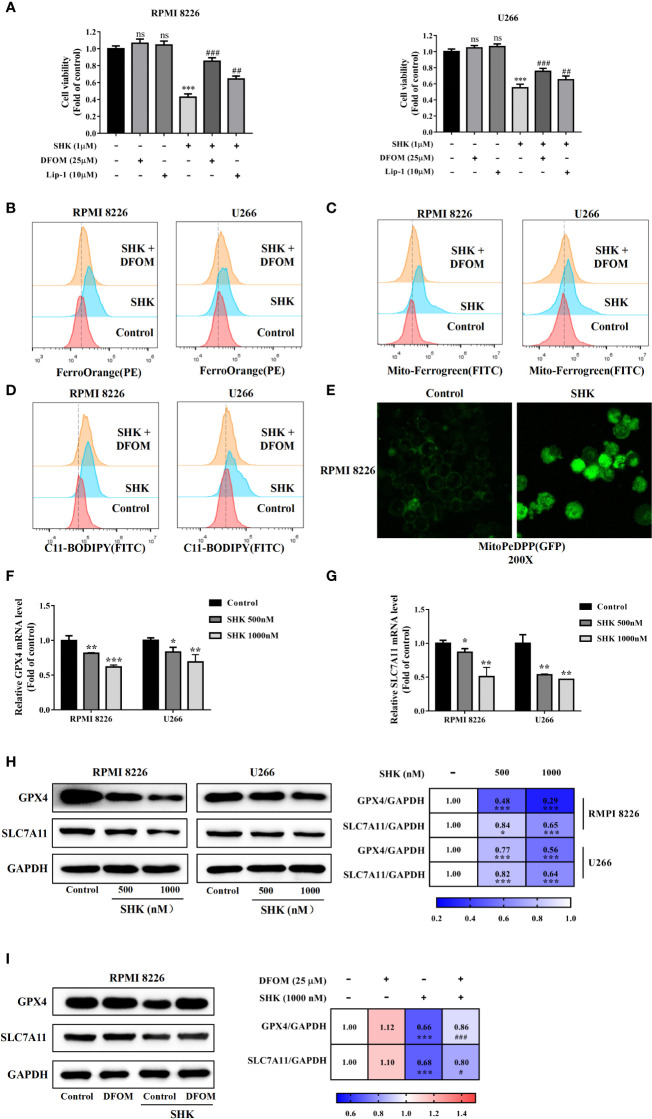
SHK facilitated MM cell ferroptosis *in vitro*. **(A)** The effects of SHK on the viability of MM cells in the presence or absence of DFOM and Lip-1. **(B)** FerroOrange probe was used to detect the level of Fe^2+^ in MM cells. **(C)** Mito-FerroGreen probe was to detect the level of mitochondrial Fe^2+^ in MM cells. **(D)** BODIPY 581/591 C11 probe was used to detect the level of lipid ROS in MM cells. **(E)** MitoPeDPP probe was used to detect the level of mitochondrial lipid ROS in MM cells. The effect of SHK on the transcription of GPX4 **(F)** and SLC7A11 **(G)**. **(H)** The effect of SHK on the translation of GPX4 and SLC7A11. **(I)** The effects of SHK on the protein level of GPX4 and SLC7A11 were determined in the presence or absence of DFOM. All the values are presented as the mean ± SD. ns, not significant; *p < 0.05, **p < 0.01,***p < 0.001, compared to control; ^#^p < 0.05,**^##^***p* < 0.01, **^###^***p* < 0.001, compared to SHK.

To verify SHK-induced ferroptosis, two specific inhibitors, DFOM and Lip-1, were used. The additional application of DFOM and Lip-1 alleviated this inhibitory effect of SHK on both RPMI 8226 and U266 cells (**Figure 2A**). Thereafter, we detected changes in the iron content of cells, which represent a specific ferroptosis index. The levels of intracellular and mitochondrial iron increased; meanwhile, DFOM treatment alleviated these effects (**Figures 2B, C**). We further measured the levels of lipid peroxidation, a feature of ferroptosis. Consistent with the iron levels, lipid ROS levels increased after SHK treatment, while DFOM treatment alleviated this effect (**Figure 2D**). Cellular mitochondrial lipid ROS also increased after SHK treatment (**Figure 2E**). Additionally, the ferroptosis markers *GPX4* and solute carrier family 7 member 11 (*xCT/SLC7A11*) were downregulated at both the transcriptional and translational levels after SHK treatment (**Figures 2F–H**). Furthermore, DFOM treatment reversed the SHK-induced downregulation of *GPX4* and *SLC7A11* (**Figure 2I**). These results confirmed that SHK triggered ferroptosis in MM cells.

### SHK treatment triggers immunogenic ferroptosis of MM cells

**Figure 3:**
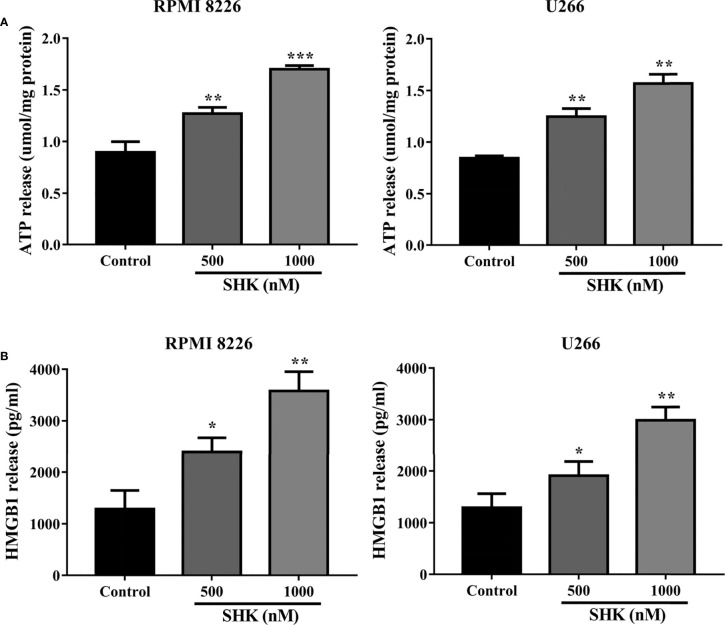
SHK Treatment triggers immunogenic ferroptosis of MM cells. **(A)** Extracellular ATP release in MM cells following different concentrations of SHK. **(B)** Extracellular HMGB1 release in MM cells following different concentrations of SHK. Data are presented as the mean ± SD. *p < 0.05, **p < 0.01, ***p < 0.001.

Immunogenic cell death (ICD), characterized by the release of ATP and HMGB1 into the extracellular space, is an essential biological process during anticancer therapy (24). Recently, the discovery of the strong immunogenicity of ferroptotic cancer cells has broadened the current concept of immunogenic cell death and opens up new possibilities for cancer treatment (25). In particular, the inducement of immunogenic ferroptosis could be beneficial for patients with cancers resistant to apoptosis and necroptosis (26). We, therefore, decided to examine the immunogenicity of ferroptotic cancer cells. As shown in **Figure 3A**, extracellular ATP release was observed in RPMI 8226 and U266 cells exposed to different concentrations of SHK. Similarly to the observed release of extracellular ATP, HMGB1 was secreted from MM cells into the extracellular space (**Figure 3B**). These results indicated that SHK treatment leads to the immunogenic ferroptosis of MM cells.

### GOT1 is a crucial factor in shk-induced ferroptotic death of MM cells

**Figure 4:**
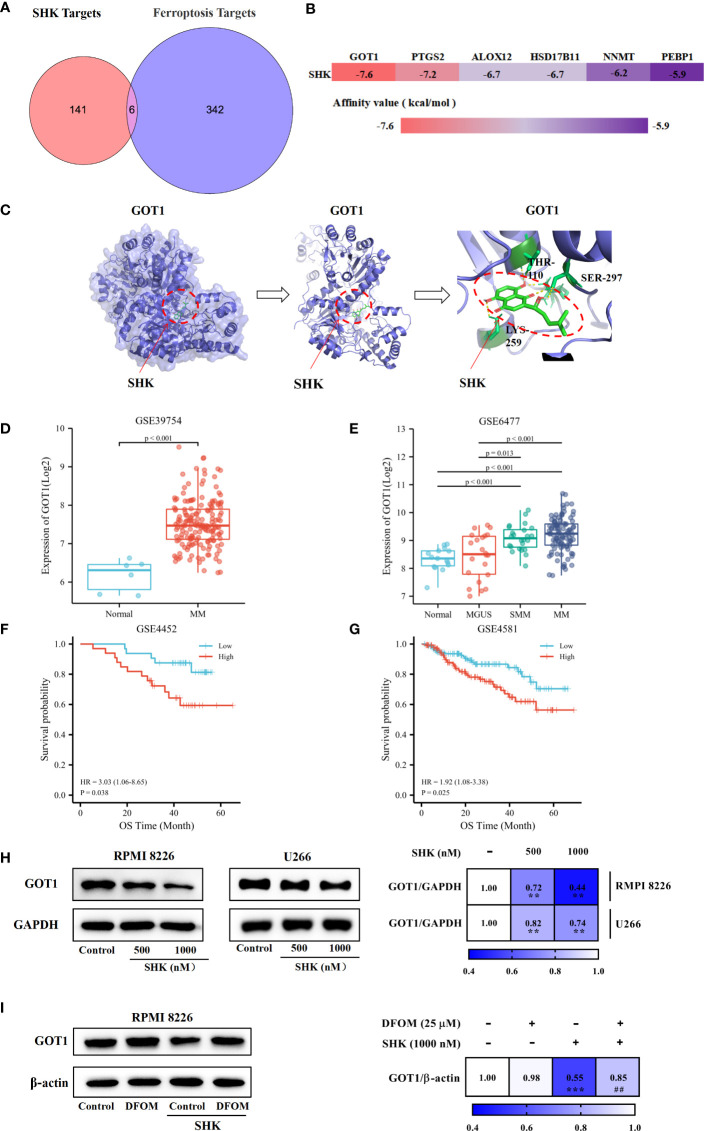
GOT1 inhibition contributes to SHK-induced ferroptosis in MM. **(A)** Common targets of SHK and ferroptosis. **(B)** The binding energy of SHK bind with potential targets. **(C)** Molecular docking between SHK and GOT1. **(D)** The comparative study of GOT1 expression between healthy people and MM patients in GSE39754 database. **(E)** The comparative study GOT1 expression among healthy people and MGUS, SMM and MM patients in GSE6477 database. **(F, G)** The K-M survival curves analysis of GSE4452 and GSE4581 databases. **(H)** The expression of GOT1 influenced by SHK treatment for 24 h in RPMI 8226 and U266 cell lines. **(I)** The effects of DFOM on SHK regulated GOT1 expression. **p < 0.01, ***p < 0.001, ##p < 0.01.

We obtained the targets of SHK using PharmMapper. Ferroptosis-related genes were obtained from the FerrDb database. The analysis of the component-, target-, and ferroptosis-related gene map revealed six candidate genes, *GOT1*, arachidonate 12-lipoxygenase (*ALOX12*), prostaglandin-endoperoxide synthase 2 (*PTGS2*), phosphatidylethanolamine binding protein 1 (*PEBP1*), nicotinamide N-methyltransferase (*NNMT*), and hydroxysteroid 17-beta dehydrogenase 11 (*HSD17B11*) (**Figure 4A**). SHK was docked with the candidate genes individually; it is generally considered that lower binding energy results in a higher affinity and binding property (**Figure 4B**). GOT1, with the lowest affinity value, may be the most effective target. Additionally, hydrogen bonds between Thr110, Ser297, and Lys259 in GOT1 with SHK were predicted (**Figure 4C**). Furthermore, we investigated the overexpression of *GOT1* in patients with MM *via* comparative analysis of the GSE39754 database (**Figure 4D**). Subsequent analysis of the GSE6477 database demonstrated that the levels of GOT1 were different among healthy individuals and patients with MGUS, SMM, or MM (**Figure 4E**). K-M survival analysis indicated that patients with MM with *GOT1*-high had a worse prognosis than those with *GOT1*-low (**Figures 4F, G**). These results show that *GOT1* might be a lethal prognosis indicator of MM. Hence, we demonstrated the inhibitory effect of SHK on GOT1 using the Western blot analysis. The results showed that SHK treatment inhibited GOT1 expression in both RPMI 8226 and U266 cells in a dose-dependent manner (**Figure 4H**). This inhibitory effect was attenuated by DFOM (**Figure 4I**). Together, these findings indicate that *GOT1* inhibition contributes to the SHK-induced ferroptosis in MM cells.

### SHK-induced GOT1 inhibition primes for ferroptosis by promoting ferritinophagy

**Figure 5:**
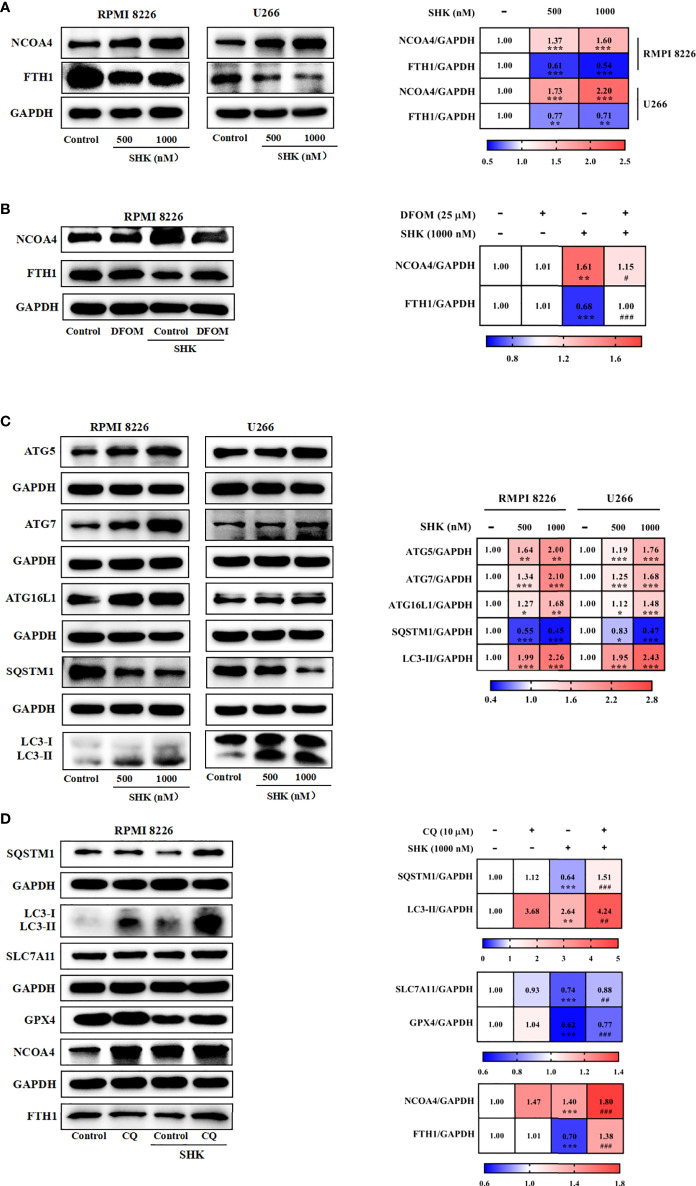
GOT1 inhibition primes for ferroptosis by promoting ferritinophagy. **(A, B)** The effects of SHK on protein level of FTH1 and NCOA4 were determined in the presence or absence of DFOM. **(C)** The protein level of ATG5, ATG7, ATG16L1, SQSTM1 and LC3B was examined after treatment with SHK for 24 h. **(D)** The effects of SHK on protein level of SQSTM1, LC3B, SLC7A11, GPX4, NCOA4 and FTH1 were determined in the presence or absence of CQ. Data are presented as the mean ± SD. *p < 0.05, **p < 0.01, ***p < 0.001, compared to control; **^##^***p* < 0.01, **^###^***p* < 0.001, compared to SHK.

Previous studies have reported that ferritinophagy is increased in response to GOT1 inhibition (9). Hence, we assessed the effect of SHK-induced *GOT1* inhibition on ferritinophagy in the two cell lines. Results showed that SHK treatment enhanced the expression of *NCOA4*, a ferritinophagy marker gene, and reduced that of *FTH1* (**Figure 5A**). DFOM ameliorated the effect of SHK treatment (**Figure 5B**). Ferritinophagy is a selective mode of autophagy; thus, we detected the levels of autophagy-related genes (ATGs). The results showed that *ATG5*, ATG7, *ATG16L1*, and *LC3B*-II were upregulated, and *SQSTM1* was downregulated upon SHK treatment (**Figure 5C**). Furthermore, we used the autophagy inhibitor CQ and found that it mitigated the changes in *SQSTM1*, *LC3B-II*, *SLC7A11*, *GPX4*, *NCOA4*, and *FTH* expression induced by SHK treatment (**Figure 5D**). These results indicated that SHK-induced *GOT1* inhibition primes ferroptosis by promoting ferritinophagy.

### SHK exerts anti-MM effects by inducing ferroptosis in tumor cells *in vivo*

**Figure 6:**
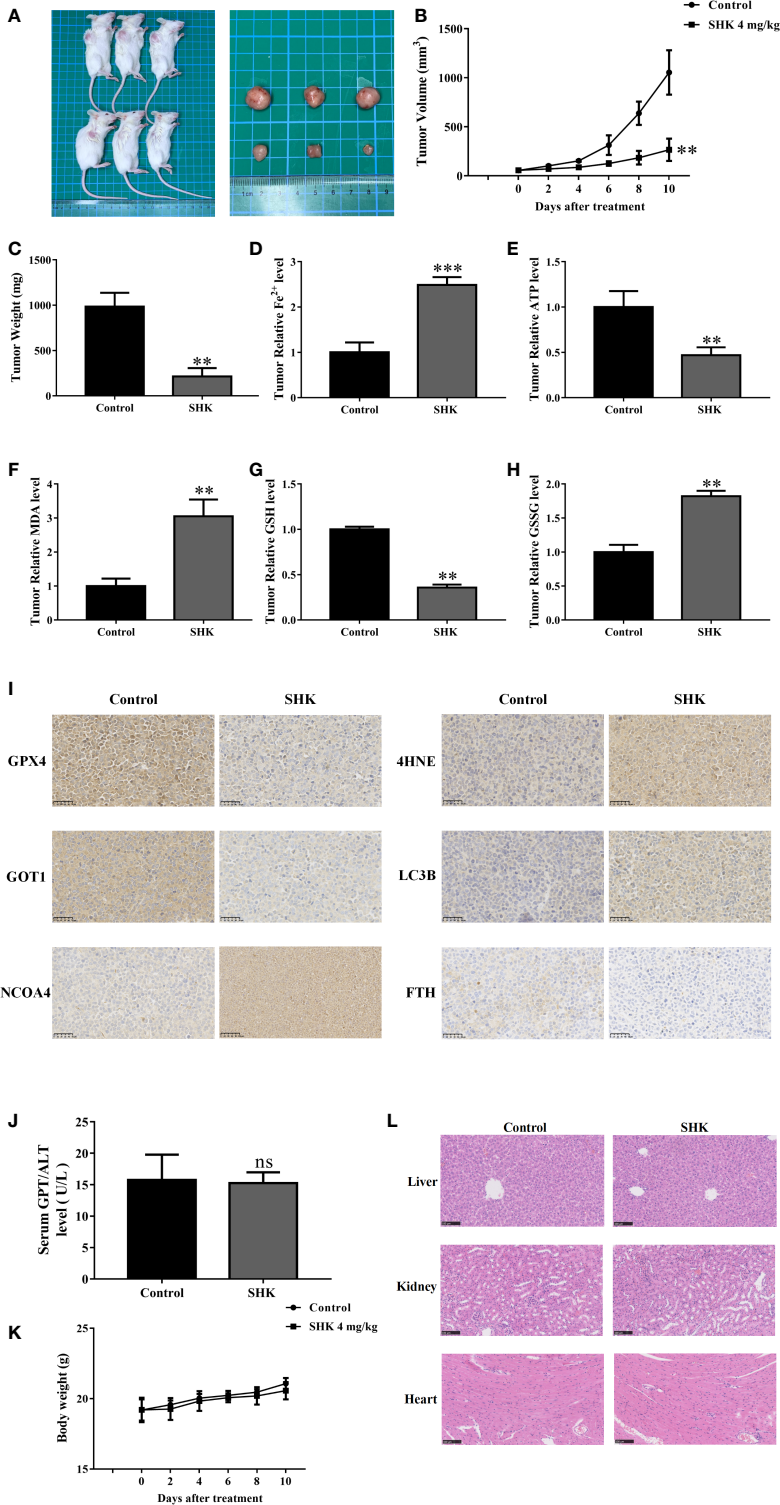
SHK results in ferroptosis induction and exerts antitumor efficacy *in vivo*. **(A)** Representative image of RPMI 8226 xenograft tumors after SHK treatment. **(B)** Tumor volume in each group. **(C)** Tumor weight in each group. **(D)** Tumor Fe^2+^ in each group. **(E)** Tumor ATP level in each group. **(F)** Tumor MDA level in each group. **(G)** Tumor GSH level in each group. **(H)** Tumor GSSG level in each group. **(I)** IHC staining were used to assess GPX4, 4HNE, GOT1, NCOA4, FTH1 and LC3B levels in tumor tissues. **(J)** Serum ALT in each group. **(K)** Body weight in each group. **(L)** HE stain in liver, kidney and heart of each group. Data are presented as the mean ± SD. ns, not significant; **p < 0.01, ***p < 0.001.

Next, we determined the therapeutic potential of SHK in a human tumor xenograft mouse model. Compared with those in the control groups, tumors in the SHK-treated mice group were small (**Figure 6A**) and showed a remarkable reduction in growth (**Figure 6B**). Meanwhile, tumor weight was reduced in the SHK treatment group (**Figure 6C**). Furthermore, SHK treatment increased the levels of Fe^2+^, MDA and GSSG (**Figures 6D, F, H**), while decreased levels of ATP and GSH (**Figures 6E, G**). We also investigated the expression of *GPX4*, *4-HNE*, *GOT1*, *NCOA4*, *FTH*, and *LC3B* in tumor tissues using IHC staining. As shown in **Figure 6I**, the expression of *GPX4*, *GOT1*, and *FTH* was inhibited by SHK treatment. Conversely, the levels of *4-HNE*, *NCOA4*, and *LC3B* increased. These findings are consistent with the results of our *in vitro* study. Notably, the changes in serum alanine transaminase (ALT) levels and body weights between the two mice groups were not statistically different (**Figures 6J, K**). Minor toxicity and side effects are among the drawbacks of this therapeutic medicine. Hence, we also evaluated the toxic effects of SHK on the livers, kidneys, and hearts of model mice. No notable dysfunctions or pathological changes were observed (**Figure 6L**).

## Discussion

MM is a hematological malignancy characterized by the infinite clonal expansion of B cell-derived plasma cells in the bone marrow cavity (27). The emergence of new drugs has broadened the range of treatment options for MM and provided a more personalized approach for relapsed/refractory patients (1, 28). Unfortunately, chemoresistance and recurrence remain major problems in clinical management (29). Therefore, it is imperative to identify new therapeutic targets and pharmaceuticals.

In this study, we observed an anticancer effect of SHK on MM cells exposed to oxidative stress. Furthermore, we found that treatment with a ferroptosis inhibitor protected MM cells against SHK-induced death, indicating the occurrence of ferroptosis. Accordingly, we determined the ferroptosis indices in MM cells during SHK treatment. As expected, iron accumulation and lipid peroxidation were triggered following SHK treatment. This finding indicates that ferroptosis contributes to the SHK-induced death of MM cells. Emerging evidence has implicated ferroptosis in the activation of antitumor immunity. During the early stages of ferroptosis, cancer cells have been shown to release damage-associated molecular patterns, stimulating immunogenicity. In our study, we explored the immunogenicity of ferroptotic tumor cells. We found that ferroptotic MM cells released ATP and HMGB1, indicating that SHK treatment triggers immunogenic ferroptosis. To explore how SHK induces ferroptosis, we used PharmMapper and found that SHK might target GOT1, and GOT1 inhibition contributes to the SHK-induced ferroptosis in MM. We further observed that the SHK-induced GOT1 inhibition promoted the release of autophagic labile iron. All *in vivo* results were consistent with those of the *in vitro* studies.

Ferroptosis is a newly recognized form of cell death (30). Ferroptosis inducers have been shown to kill tumor cells effectively. Several drug-resistant tumor cells, especially those with mesenchymal and dedifferentiated features, are sensitive to ferroptosis (31–33). Additionally, ferroptosis-inducing agents combined with chemotherapeutic drugs can enhance anticancer efficacy (34, 35). A previous study showed that free fatty acids (FFAs) released by adipocytes have a double effect on MM cells. Lower concentrations of FFAs increase MM cell proliferation. Higher concentrations induce lipotoxicity in MM cells. Lipotoxicity occurs *via* the ferroptosis pathway (36). Ferroptosis may also contribute to the anti-MM effects of apigenin, fingolimod, *Fumaria officinalis* extracts, *Thymus vulgaris* extracts, and *Arctium lappa* extracts (37–39; 40). In U266, MM.1S, OPM-2, and H929 MM cell lines, the iron-bortezomib combination reportedly induces persistent lipid damage and triggers cell death more effectively compared to individual treatment (41). Supplementation of iron with a bortezomib-melphalan-prednisone regimen increases the therapeutic response and prolongs remission *in vivo* (41). These findings suggest that induction of ferroptosis in MM cells is a promising strategy for MM therapy.

Although ferroptosis-related phenomena have been observed in cells following SHK treatment, researchers have not integrated these phenomena into a unified process. ROS overproduction has been observed in melanoma, glioma, nasopharyngeal carcinoma, cholangiocarcinoma, colon cancer, and primary effusion lymphoma cells after SHK treatment (42–44; 45; 46). GSH depletion and SLC7A11 downregulation have been observed in glioma cells after SHK treatment (47). To our knowledge, this is the first study to show that SHK can induce ferroptosis.

GOT1 is overexpressed in many cancers; thus, it has been identified as a potential therapeutic target (11). GOT1 contributes to cancer cell proliferation, migration, and invasion in pancreatic ductal adenocarcinoma and colorectal cancer (48–50). Meanwhile, GOT1 overexpression reduces the sensitivity of colorectal cancer cells to 5-fluorouracil (5-FU) and triple-negative breast cancer cells to doxorubicin (51, 52). An increased GOT1 expression predicts an adverse prognosis in acute myeloid leukemia (53). We demonstrated for the first time that GOT1 is a poor prognostic biomarker of MM.

The role of GOT1 in ferroptosis has been the subject of previous studies on several other diseases. Daniel et al. reported that GOT1 inhibition promotes autophagic labile iron release to induce ferroptosis in pancreatic ductal adenocarcinoma, which is consistent with our data (9). However, some of our data contrasts with several previous studies; for example, Wei etal. (54) reported that sepsis increases the expression of GOT1 to promote ferroptosis, thus exacerbating sepsis-associated encephalopathy. Zhang et al. reported that miR-9 suppresses the activity of GOT1 to inhibit ferroptosis in melanoma (55). The differences in these findings may be associated with genotypes, nutrient environment, tissue of origin, or autonomous metabolism.

**Figure 7:**
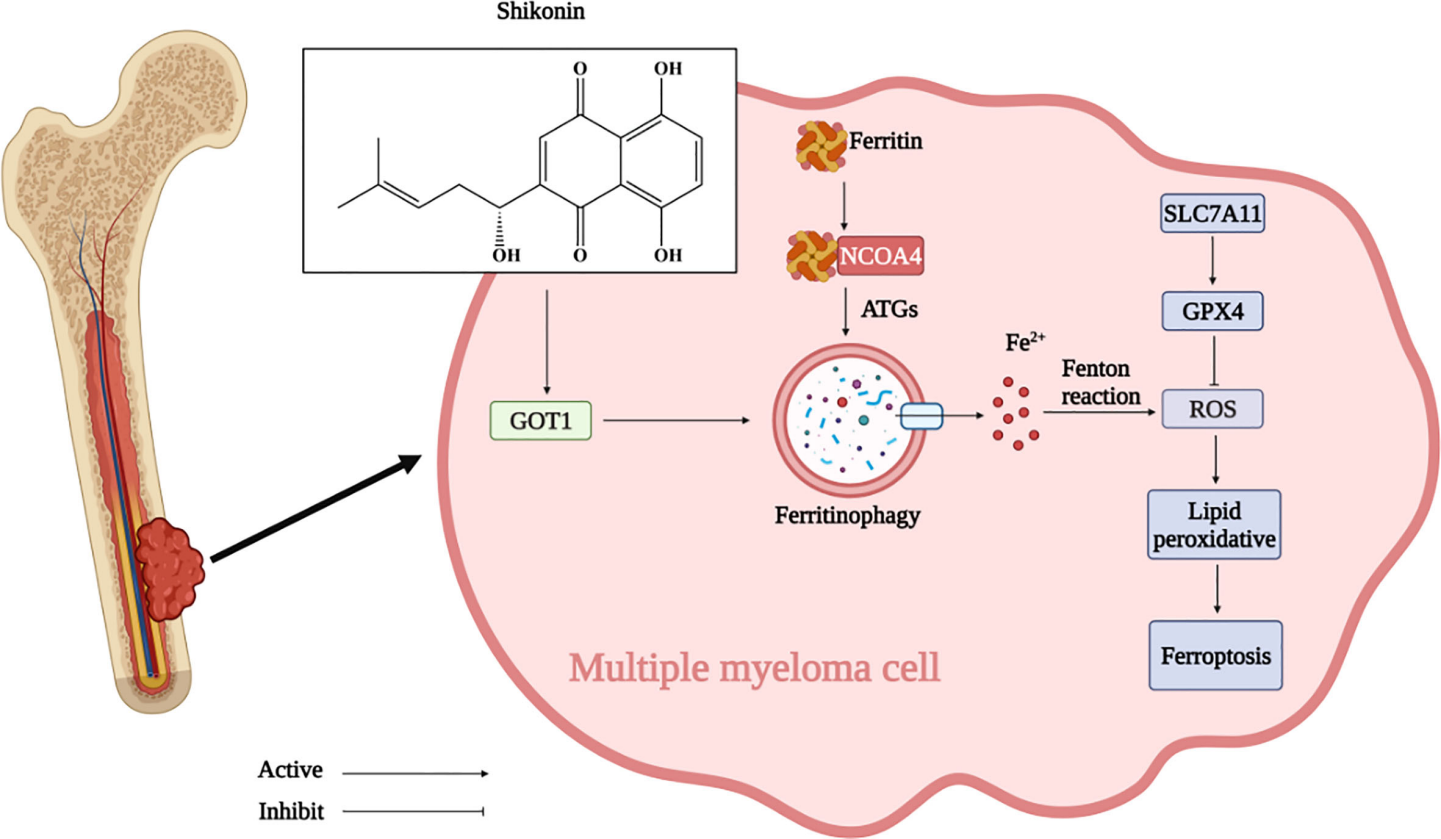
SHK inhibited MM development by regulating GOT1 to activate ferritinophagy and further to promote ferroptosis in MM. (Created with BioRender.com).

In summary, our study showed the anti-cancer effect of SHK against MM *in vivo* and *in vitro*. Moreover, this study is the first to provide evidence that SHK induces ferroptosis in MM by *GOT1*-mediated ferritinophagy (**Figure 7**). We highlight the potential value of SHK as an effective candidate for MM treatment and a ferroptosis inducer. However, whether SHK could be an effective therapeutic option for patients with MM is also unclear. Therefore, our future work involves the study of the full anti-MM potential of SHK.

## Data availability statement

The datasets presented in this study can be found in online repositories. The data has been deposited in the GEO repository, and can be found using accession numbers GSE39754, GSE6477, GSE4452, and GSE4581.

## Ethics statement

The animal study was reviewed and approved by Animal Ethical and Welfare Committee of Zhejiang Chinese Medical University.

## Author contributions

WL, HF, ZC, and SQ conceived and designed the experiments. WL, HF, LF, HC, and TG performed laboratory experiments. WL, HC, and SQ drafted the manuscript. All authors contributed to the article and approved the submitted version.

## Funding

This study was supported by the Postgraduate Scientific Research Fund Project of Zhejiang Chinese Medical University (2021YKJ11), the Zhejiang Science and Technology Innovation Program for college students (2022R410A035), and the Hangzhou Health Science and Technology Project (Z20210039).

## Acknowledgments

We would like to thank Editage (www.editage.cn) for English language editing.

## Conflict of interest

The authors declare that the research was conducted in the absence of any commercial or financial relationships that could be construed as a potential conflict of interest.

## Publisher’s note

All claims expressed in this article are solely those of the authors and do not necessarily represent those of their affiliated organizations, or those of the publisher, the editors and the reviewers. Any product that may be evaluated in this article, or claim that may be made by its manufacturer, is not guaranteed or endorsed by the publisher.
